# Effects of Developmental Programming Caused by Maternal Nutrient Intake on Postnatal Performance of Beef Heifers and Their Calves

**DOI:** 10.3390/ani9121072

**Published:** 2019-12-03

**Authors:** Agustí Noya, Isabel Casasús, Javier Ferrer, Albina Sanz

**Affiliations:** Department of Animal Production, Centro de Investigación y Tecnología Agroalimentaria (CITA) de Aragón, Instituto Agroalimentario de Aragón, IA2 (CITA-Universidad de Zaragoza), 50059 Zaragoza, Spain; anoya@cita-aragon.es (A.N.); icasasus@cita-aragon.es (I.C.); jferrerac@aragon.es (J.F.)

**Keywords:** puberty, fertility, metabolic and endocrine profile, early pregnancy, malnutrition, long-term effects

## Abstract

**Simple Summary:**

The nutrient intake of a cow during the early stages of pregnancy can have detrimental effects on the developing fetus. The adaptive response of the fetus to the restricted intrauterine environment can modify organ development. In this study, we assessed the consequences of maternal undernutrition in the first third of gestation on female offspring during its rearing, gestation, and lactation periods. We confirmed that maternal nutrient restriction decreased the heifer live weight at weaning (four months old); this difference disappeared at puberty, the end of rearing, and during the following gestation and lactation periods. Consequently, heifers from nutrient-restricted dams had impaired metabolic status around the onset of puberty. Maternal undernutrition reduced the number of antral follicles at breeding (16 months old); however, it did not affect the pregnancy rate after a single artificial insemination nor the calf weight gains during the first lactation period. In conclusion, early maternal subnutrition had long-term effects on heifer postnatal performance during the first four months of life which compromised heifer metabolic status during rearing but did not affect the reproductive performance during its first gestation and lactation periods.

**Abstract:**

In this study, we evaluated the effects of maternal subnutrition in early pregnancy on the growth and reproductive performance of female offspring during their rearing, first gestation, and lactation. We inseminated 21 Parda and 15 Pirenaica multiparous cows and assigned them to a CONTROL (100% of nutrition requirements) or SUBNUT (65%) diet until day 82 of gestation. Cows were fed 100% requirements afterward. During the rearing of female offspring, growth, physiological profiles and ovarian follicular dynamic were studied. At 16 months old, heifers were inseminated. After first calving, dam–calf weights were recorded during lactation. Heifers born from CONTROL cows were heavier at weaning (four months old) than heifers born from SUBNUT cows, but this difference disappeared at the end of rearing and during the first gestation and lactation periods. All heifers reached puberty at a similar age and live weight. During rearing, SUBNUT heifers had higher concentrations of non-esterified fatty acids, urea, and cholesterol and a lower antral follicle count than CONTROL, but no difference was found in their fertility rate. After heifer first calving, dam–calf weights were similar among groups. In conclusion, maternal undernutrition reduced offspring postnatal gains at weaning, compromising metabolic status and follicle population during rearing but did not impair performance in the first gestation and lactation periods of beef heifers.

## 1. Introduction

Beef cattle production systems have increasingly adapted to extensive conditions to reduce feed costs. Animals are managed during long periods on grazing systems, where food availability depends exclusively on pastures. Many factors can influence the quality and quantity of this nutrient source during the cow–calf production cycle. Restricted nutrition can occur during early gestation when critical processes such as embryonic cell lineage allocation and maternal recognition of pregnancy take place. The nutritional microenvironment in ovaries and reproductive tract induces cellular and molecular alterations in the peri-implantation embryo to adapt its physiology to a poor environment, modifying the establishment of founder cell lineages or gene activation [1,2]. This developmental strategy can produce adverse embryo and/or fetal programming, resulting in offspring with a higher risk of developing deleterious phenotypes in adulthood [1,3]. Through epigenetic mechanisms, maternal nutrition is linked to offspring phenotype as a consequence of the organism memory of past metabolic and environmental events [4,5].

In general, most studies established that the consequences of feed restriction during early pregnancy on progeny growth can be alleviated by adequate nutrition in late pregnancy with few consequences on postnatal growth [6,7,8]. However, the impact of gestational undernutrition, either in early, mid or late gestation, on the reproductive performance of the progeny is not fully understood. Several studies reported negative effects on reproductive traits of the female offspring, with lower antral follicle counts [9], impaired ovulation rate [10] or decreased fertility [10,11]. In contrast, other studies described equal [12] or higher [13] numbers of antral follicles and higher ovulation rate [13] in females from nutrient-restricted dams. To explain these contradictory results, Smith et al. [13] suggested that the effects of maternal nutrient restriction depends on the degree and timing of this restriction and the post-restriction diet. In cattle, few studies have evaluated the consequences of embryo and/or fetal programming in a poor uterine environment on heifer reproductive performance and whether these potential effects can be devolved to heifer offspring which is important to consider when designing feeding practices in the beef cattle husbandry. Micke et al. [14] indicated that a refining nutritional program during early gestation may optimize production objectives in the progeny.

These effects could vary depending on the breed and genetic background. Metabolic and endocrine responses to management can differ in the function of the breed [15,16,17]. In this study, Parda de Montaña (PA) and Pirenaica (PI) animals were used, because they are the two main beef cattle breeds adapted to a semi-extensive system of animal husbandry in the Pyrenees mountain region in Northern Spain.

Our previous results highlighted that dam nutrition in early pregnancy had long-term effects on offspring growth during the first four months of life, with this physiological evidence being more pronounced in PI cow–calf pairs [18]. In the current study, we hypothesized that poor maternal nutrition could have a residual impact on postnatal performance of beef heifers during rearing, first gestation, and lactation periods. We aimed to evaluate the effects of adverse prenatal environment caused by maternal energy intake on postnatal growth, metabolism, and reproduction of PA and PI heifers and on their offspring weights during their first lactation.

## 2. Materials and Methods

**Figure 1:**
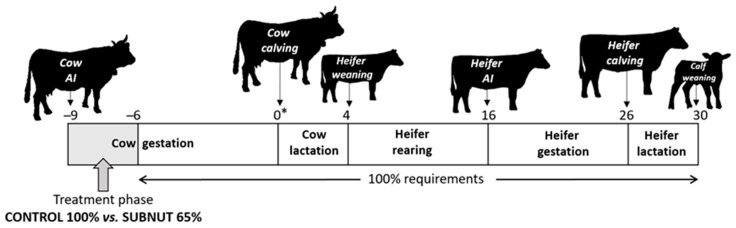
Experimental design. * month; CONTROL, cows fed 100% of their nutritional requirements during the first third of pregnancy; SUBNUT, cows fed 65% of their nutritional requirements during the first third of pregnancy; AI, artificial insemination.

All the procedures were approved by the Animal Ethics Committee of the Centro de Investigación y Tecnología Agroalimentaria (CITA) of Aragón, Spain. The care and use of animals were performed in accordance with the guidelines of the European Union (Directive 2010/63/EU) regarding the protection of animals used for experimental and other scientific purposes [19]. Gestation and lactation phases of this study were conducted at CITA-La Garcipollera Research Station (the mountain area of the central Pyrenees, Huesca, Spain, 945 m above sea level (a.s.l.)), and the rearing phase at CITA-Montañana Research Station (Zaragoza, Spain, 225 m a.s.l.). The experimental design of this study is shown in Figure 1.

### 2.1. Management during Cow Gestation (Maternal Nutrition Treatment) and Lactation

We synchronized 53 PA and 32 PI multiparous cows which were then artificially inseminated (AI) and distributed into two nutrition treatments from the day of AI, both fed a total mixed ration (alfalfa hay 25.0%, cereal straw 25.0%, crushed barley 25.0%, dehydrated alfalfa 10%, rapeseed meal 6.5%, citrus pulp 4.5%, soybean meal 2.5%, and vitamin–mineral complex 1.5%; Table 1) during the first third of gestation as described in Noya et al. [15]. Briefly, the control group (CONTROL, 574 ± 8.9 kg live weight (LW); 2.80 ± 0.038 body condition score (BCS) on a 5 point scale [20]) was fed a diet that supplied 100% of the estimated energy requirements for cow maintenance, lactation, and gestation; and the nutrient-restricted group (SUBNUT, 568 ± 7.6 kg LW; 2.86 ± 0.032 BCS) received 65% of their requirements. After this treatment phase, the CONTROL group maintained its LW and BCS (583 ± 8.6 kg LW; 2.90 ± 0.040 BCS), whereas they decreased in SUBNUT animals (538 ± 7.2 kg LW; 2.65 ± 0.033 BCS, *p* < 0.001), reported by Noya et al. [21]. All dams were fed 100% of the requirements during the remainder of gestation and the next lactation, using the same total mixed ration described above. Feed was provided at 08:00 and cows were tied up for a maximum of 2 h until they finished the restricted amount assigned to each. Calves’ diets consisted exclusively of maternal milk in a restricted twice-daily nursing system. Calves were weaned at the age of 120 days.

### 2.2. Heifer Management during Rearing

After calf weaning, only female calves were selected for this study. We distributed 21 PA and 15 PI heifers into two groups, according to their maternal nutrition: heifers from CONTROL cows (CONTROL heifers, *n* = 17 (15 PA + 2 PI)) and heifers from SUBNUT cows (SUBNUT heifers, *n* = 19 (6 PA + 13 PI)). Four-month-old heifers were housed in a pen and fed 2 kg/head/day of a commercial concentrate (corn 47%, corn gluten feed 15%, barley 15%, soya flour 6%, sunflower pulp 6%, carob flour 4%, palm oil 4%, and vitamin–mineral complex 3.2%) plus ad libitum meadow hay and barley straw (Table 1) during rearing. The meadow hay intake was recorded (offer minus refusal) per group (CONTROL versus SUBNUT), and their values are expressed as mean values.

Sixteen-month-old heifers were synchronized to estrus with a protocol based on a progesterone-releasing intravaginal device (PRID Delta 1.55 g, CEVA, Libourne, France) and a 100 μg injection of gonadotropin-releasing hormone (GnRH, Cystoreline, CEVA, Libourne, France), followed 7 days later by a 25 mg injection of prostaglandin F2α (Enzaprost T, CEVA, Libourne, France). After 9 days, the PRID was removed and 250 IU of pregnant mare serum gonadotropin (Foligon, Intervet International B.V., Boxmeer, The Netherlands) was administered, followed 48 h later by a second injection of GnRH (100 μg). Eight hours after the second GnRH injection, heifers were inseminated with a PI sire of proven fertility by an expert technician.

#### 2.2.1. Heifer Growth during Rearing

Heifers were weighed monthly during rearing (from 4 to 16 months of age) to calculate the average daily gain (ADG) by linear regression. The body development of the heifers was studied by recording their size measures at 4 (weaning), 12, and 16 months of life (AI). The variables assessed were height at withers (distance from the floor to the highest point of the withers), hearth girth (circumference immediately behind the shoulder blades in a plane perpendicular to the body axis), rump width (maximum distance between iliac tuberosities), and rump length (distance from the ischial tuberosity to the external iliac tuberosity). The external pelvic area was estimated as the product of rump width and rump length [22].

#### 2.2.2. Heifer Reproductive Performance during Rearing

To determine the onset of puberty, progesterone concentration was assessed from 8 to 16 months of age (AI). Samples were collected by coccygeal venipuncture into heparinized tubes (BD Vacutainer Becton-Dickenson and Company, Plymouth, UK) on days 1 and 10 every 28 day period [23]. Samples were centrifuged at 1500× *g* for 20 min at 4 °C immediately after collection, and the plasma was harvested and frozen at −20 °C until analysis. Plasma progesterone concentration (ELISA test, sensitivity: 0.27 ng/mL) was measured using a specific kit for cattle (Ridgeway Science, Lydney, UK). The mean intra-assay and inter-assay coefficients of variation were 8.0% and 10.4%, respectively. The age at puberty was defined as the date of collection of the first sample that contained ≥1 ng/mL of plasma progesterone. The mature LW of the heifers was established at 580 kg for both breeds [24] to calculate the percentage of mature LW at which heifers reached puberty. Follicle population, corpus luteum, and ovary size were recorded at 9.5, 13.0, and 15.5 months of life by ultrasonography (Aloka SSD-500V, Aloka, Madrid, Spain) using a linear-array 7.5 MHz transducer. The total number of follicles per pair of ovaries was recorded in three categories: small (<5 mm in diameter), medium (between 5 and 10 mm in diameter), and large follicles (>10 mm in diameter) [25]. The diameters of the dominant follicles, corpus luteum, and the ovaries were assessed by considering the average between measurements of their two perpendicular axes [26]. Pregnancy diagnosis was performed by ultrasonography on day 37 post-AI to assess the pregnancy rate to single AI.

#### 2.2.3. Heifer Metabolic and Endocrine Profiles during Rearing

To assess the metabolic and endocrine status of heifers during rearing, blood samples were collected every three months into EDTA or heparinized tubes to determine glucose, non-esterified fatty acids (NEFAs), urea, cholesterol, and insulin-like growth factor 1 (IGF-1) concentrations. Furthermore, in the case of IGF-1, blood samples were previously taken every month from their mothers during the first third of gestation (cow gestation, when the maternal nutritional treatment was applied) and from heifers during their suckling period (cow lactation [18]). Blood samples were centrifuged at 1500× *g* for 20 min at 4 °C, and plasma samples were taken and frozen at −20 °C until analysis. An automatic analyzer (GernonStar, RAL/TRANSASIA, Dabhel, India) was used to measure blood concentrations of glucose (glucose oxidase/peroxidase method, sensitivity: 0.056 mmol/L), urea (kinetic UV test, sensitivity: 0.170 mmol/L), and cholesterol (enzymatic colorimetric method, sensitivity: 0.026 mmol/L). The mean intra-assay and inter-assay coefficients of variation for these molecules were <5.4% and <5.8%, respectively. Non-esterified fatty acids (NEFAs, enzymatic method, sensitivity: 0.06 mmol/L) were analyzed using a commercial kit (Randox Laboratories Ltd., Crumlin Co., Antrim, UK). The mean intra-assay and inter-assay coefficients of variation were 5.1% and 7.4%, respectively. Insulin-like growth factor 1 (enzyme immunoassay, sensitivity: 20 ng/mL) was determined using a solid-phase enzyme-labeled chemiluminescent immunometric assay (Immulite, Siemens Medical Solutions Diagnostics Limited, Llanberis, Gwynedd, UK). The mean intra-assay and inter-assay coefficients of variation were 3.1% and 12.0%, respectively.

### 2.3. Heifer Management during Gestation and First Lactation

From AI to one month before the expected calving date, pregnant heifers grazed on mountain meadows (4 heifers/ha) following the traditional management system [24]. These pastures were primarily composed of grasses (*Festuca arundinacea, Festuca pratensis*, and *Dactylis glomerata*), legumes (*Trifolium repens*), and other species (1191 kg dry matter/ha). During this period, two heifers experienced pregnancy losses. From the last month of gestation, heifers were housed and fed 9 kg/head/day of meadow hay (Table 1). After the first calving, heifers received 10 kg/head/day during lactation of the same dry total mixed ration described above (Table 1). Heifers reared their calves until weaning at day 105. Calves had free access to suckle their dams and received no other feed during lactation period. Water and vitamin–mineral supplements (lick block) were supplied in all phases throughout the experiment.

Heifers and their calves were weighed at calving and weaning to calculate their average daily gain (ADG). The heifer body condition score (BCS) was assessed at calving by two expert technicians, based on the estimation of fat covering loin, ribs, and tail head (0 to 5 scale [20]), and calving ease was classified into two categories: assisted or unassisted. Assisted calving included all types of assistance, from manual pull to caesarean section.

### 2.4. Statistical Analyses

All statistics were calculated using SAS statistical package v.9.4 (SAS Institute Inc., Cary, NC, USA). The normal distribution of data was assessed with the Shapiro–Wilk test (*p* > 0.05). Heifer BCS at calving, ADG of heifers and calves (heifer progeny), and the age and percentage of mature LW when heifers reached puberty were analyzed with a generalized linear model (GLM procedure) with the nutritional treatment (CONTROL versus SUBNUT), breed (PA versus PI), and their interaction as fixed effects. In the case of ADG of calves (heifer progeny), sex was considered as a fixed effect. The LW of heifers and their calves (heifer progeny), body size measures, follicle population, corpus luteum and ovary size, and metabolite (glucose, NEFAs, urea, and cholesterol) and hormone (IGF-1) concentration were analyzed using a mixed linear model (MIXED procedure) for repeated measures based on Kenward–Roger’s adjusted degrees of freedom solution. The fixed factors were nutritional treatment, breed, and their interactions as the between-subject effects, sampling day as the within-subject effect, and animal as the random effect (experimental unit). In the case of LW of calves (heifer progeny), sex was considered as a fixed effect. The least squares (LS) means of the treatments were estimated per fixed effect, and pair-wise comparisons of the means were obtained by the probability of difference (PDIFF) option in the LS means procedure. Fertility rate, percentage of pubertal heifers at 12 and 16 months of age, calving assistance, and male/female calf ratio were assessed using the F-test (FREQ procedure). Relationships among the studied parameters were determined using Pearson’s correlation coefficients. The level of significance for all tests was *p* < 0.05. The results are presented as LS means ± standard error (SE) in the text or with the residual standard deviation (RSD) in the tables.

## 3. Results

No significant interactions between maternal nutrition and breed were found in our results throughout the study; thus, the results were examined separately.

### 3.1. Heifer Growth during Rearing

The CONTROL heifers were heavier than the SUBNUT heifers at weaning (*p* = 0.020). These differences disappeared thereafter, and both groups had similar LW at AI (Table 2). During rearing, SUBNUT heifers had higher ADG than CONTROL heifers, but this difference was not significant (*p* > 0.05). Regarding the breed, despite no differences being found among breeds at weaning (*p* > 0.05), PA had higher LW at AI than PI heifers (*p* = 0.036) which reflected the higher weight gains during rearing in PA than in PI heifers (*p* = 0.002).

Regarding heifer growth, body size was not influenced by maternal nutrition (Table 2). No differences were found in any size trait during rearing between CONTROL and SUBNUT heifers (*p* > 0.05). Inter-breed differences were evidenced from 12 months of age onward, since PA heifers had higher values than PI in terms of height at withers, heart girth, and external pelvic area, both at 12 and 16 months of age (*p* < 0.05). The heart girth during rearing was highly correlated with the heifer LW (*r* = 0.99, *p* < 0.001), with a good predictive capacity (y = 4.67x − 401.23, *r^2^* = 0.98, where y = LW and x = heart girth).

During rearing, the average hay intake was 5.36 kg DM/day for CONTROL and 4.57 kg DM/day for SUBNUT heifers.

### 3.2. Heifer Reproductive Performance during Rearing

All heifers reached puberty at a similar age (12.0 ± 1.6 months) and LW (340 ± 30.3 kg, which was 59% of expected mature LW), regardless of the maternal nutrition or breed (*p* > 0.05, Table 3). Of the heifers, 56% were pubertal at 12 months (average age at puberty) and 91% had reached puberty at 16 months. Heifers were inseminated at 16 months of age (average LW 408 kg, which was 70% of expected mature LW). Heifer age at puberty was negatively correlated with its LW at weaning 8 months before (*r* = −0.44, *p* = 0.001).

Regarding ovarian follicle population (Table 4), at 9.5 months of age, SUBNUT heifers had more medium follicles than CONTROL heifers (*p* = 0.019). At 13 months of age, CONTROL heifers had more large follicles than SUBNUT heifers (*p* = 0.041), and at 15.5 months of age, CONTROL heifers had more small follicles (*p* = 0.011) and less large follicles (*p* = 0.032) than SUBNUT heifers. No differences were found in the presence or absence of a corpus luteum, in its diameter, or in the ovary size between CONTROL and SUBNUT heifers at either age (*p* > 0.05). Regarding breed, PA had more large follicles (*p* = 0.044) and larger ovary diameter (*p* = 0.009) than PI heifers at 9.5 months of age. At 15.5 months of age, the diameter of the dominant follicle (*p* = 0.017) and the ovary diameter (*p* = 0.003) were higher in PA than in PI heifers. The ovary diameter at 15.5 months was positively correlated with the heart girth of heifers at weaning 11.5 months before (*r* = 0.64, *p* < 0.001).

The mean fertility rate to a single AI was 80%, regardless of maternal nutrition or breed (*p* > 0.05, Table 3).

### 3.3. Heifer Metabolic and Endocrine Profiles during Rearing

**Figure 2:**
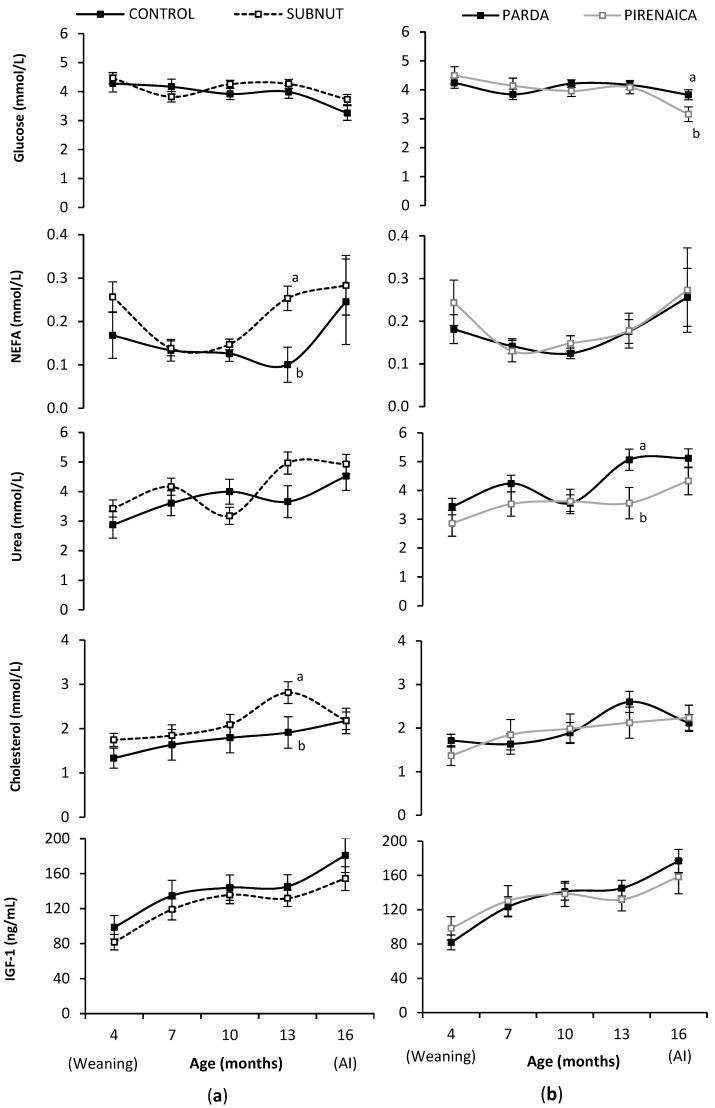
Heifer metabolic and endocrine profiles during rearing according to (**a**) maternal nutrition and (**b**) breed. CONTROL, heifers from cows fed 100% of their requirements in early pregnancy; SUBNUT, heifers from cows fed 65% of their requirements in early pregnancy; AI, artificial insemination. ^a, b^ Means at given age with different letters differ significantly (*p* < 0.05).

Metabolic and endocrine profiles of the heifers are presented in Figure 2.

Glucose concentration did not differ between CONTROL and SUBNUT heifers during rearing (*p* > 0.05), and PA had higher concentration than PI at AI (*p* = 0.036).

The SUBNUT heifers had higher NEFA concentration than CONTROL heifers at 13 months of age (*p* = 0.004), whereas no differences were found among breeds (*p* > 0.05).

Urea concentration in the SUBNUT heifers tended to be higher than in CONTROL heifers throughout rearing, specifically at 13 months (*p* = 0.053). Regarding the breed, PA heifers had higher urea concentration than PI heifers with statistical differences at month 13 (*p* = 0.027). Urea concentration was correlated with the heifer ADG during rearing (*r* = 0.65, *p* < 0.01).

The SUBNUT heifers had higher cholesterol concentrations than CONTROL heifers during rearing, with statistical differences at month 13 (*p* = 0.043). No differences were found among breeds (*p* > 0.05). A strong relationship was found between cholesterol and urea concentrations at 13 (*r* = 0.70, *p* < 0.001) and 16 months (*r* = 0.52, *p* = 0.002).

No differences were found in IGF-1 concentration between CONTROL and SUBNUT heifers, or between PA and PI heifers throughout rearing (*p* > 0.05). The mean IGF-1 concentration of heifers during rearing was correlated with their age at puberty (*r* = −0.71, *p* < 0.001) and their ADG during rearing (*r* = 0.44, *p* = 0.009). The mean IGF-1 concentration of heifers during rearing was positively correlated with the IGF-1 concentration during their lactation period (0–4 months old; *r* = 0.42, *p* = 0.013), which, in turn, was correlated with the IGF-1 concentration of their mothers during the first third of pregnancy (maternal nutrient treatment, *r* = 0.35, *p* = 0.035).

### 3.4. Heifer Performance during Gestation and First Lactation

The CONTROL and SUBNUT heifers had similar LWs and ADGs during gestation and the following lactation period (*p* > 0.05, Table 5). Similarly, no differences were found between PA and PI LWs and ADGs during gestation and the following lactation (*p* > 0.05), but PA had lower BCS at calving than PI (*p* = 0.001). No differences in the calving assistance were found between the CONTROL and SUBNUT or between PA and PI heifers (*p* > 0.05).

Regarding the calves born from heifers, the male/female calf ratio was lower in SUBNUT and in PI heifers than in CONTROL and PA heifers but not significantly (*p* > 0.05). Maternal nutrition had no effects on calf LW at birth or ADG during lactation (*p* > 0.05). Regarding breed, PA and PI calves had similar LWs at birth (*p* > 0.05), but PA calves had higher ADG than PI calves during lactation (*p* = 0.031) which implied higher LW at weaning (*p* = 0.012). Calf sex had no effect on LW at birth (36 ± 1.4 versus 33 ± 1.4 kg for male and female calves, respectively, *p* > 0.05), at weaning (103 ± 7.4 versus 111 ± 5.9 kg for male and female calves, respectively, *p* > 0.05), or on ADG during lactation (0.636 ± 0.0872 versus 0.775 ± 0.0872 for male and female calves, respectively, *p* > 0.05).

The calf ADG during lactation was correlated with its dam LW at calving (*r* = 0.65, *p* < 0.001). Calf LW at weaning was highly correlated with the LW of heifer when it was weaned (*r* = 0.61, *p* = 0.001).

## 4. Discussion

### 4.1. Growth of Heifers and Their Calves

Maternal nutrition in early pregnancy impaired the heifer LW at weaning (at the end of its suckling period); however, this difference disappeared during subsequent heifer rearing, gestation, and lactation. This result suggests that SUBNUT heifers exhibited compensatory growth to mitigate the growth delay recorded at weaning. The ADG of SUBNUT heifers during rearing was higher than that of CONTROL heifers, although not significantly. Maternal nutrition in early pregnancy most impacts on organ and tissue development, with potential long-term consequences during any postnatal age, rather than on fetal growth, which is associated with maternal nutrition during later pregnancy [27]. These long-term consequences were evidenced in the offspring endocrine profiles at birth [15], in their performance during lactation [18], and at weaning, with retarded growth in SUBNUT heifers in the current study. The similar LW during rearing, gestation, and first lactation of heifers suggested that the growth delay observed at weaning was not a permanent stunting and an adequate nutrition could overcome these LW differences, agreeing with the results reported by Freetly et al. [28]. In our study, after the first calving, the calf LW at birth and growth rate were not affected by the early nutritional treatment, suggesting that maternal nutrition did not affect the performance of this second generation, at least during lactation. However, since females of these breeds reach maturity when they are 4 to 5 years old [29], further studies are needed to determine if the heifer growth delay at weaning would impact the attainment of adult weight and mature size. Regarding breed, PA heifers had higher growth rates during rearing, BCS at calving, and calf LW at weaning than PI heifers, in accordance with previous studies [30], which evidenced similar interbreed differences.

To make management decisions considering these breed differences, heart at girth was confirmed to be a useful, objective, and easily obtainable parameter to estimate heifer LW, especially in those livestock production systems where the ability to use a scale is limited.

### 4.2. Heifer Reproductive Performance

In the current study, all heifers reached puberty at a similar age and LW, regardless of the maternal nutrition or the breed. Despite the growth delay observed at weaning in the SUBNUT group, adequate nutrition during rearing could prevent the potential consequences. Puberty is reached at a critical LW of around 55% of the mature LW, irrespective of the growth patterns [31]. Aligned with our results, Corah et al. [32] reported that age at puberty was not affected in female progeny of heifers fed a diet that met 65% of their recommended energy intake, although in that case, the energy restriction was performed during the last third of gestation. Similarly, Smith et al. [13] described no differences in age at puberty between control and maternal nutrient-restricted sheep during the first 55 days of gestation. Regarding breed, due to the higher ADG during rearing, PA heifers reached puberty 1 month earlier and 23 kg heavier than PI heifers, although these differences were not significant. In a previous study, PA and PI heifers reached puberty at a similar LW, but two months earlier than in our study due to the fact of their higher growth rates after weaning [30].

In our study, the follicular population was affected by maternal nutrition. Differences in medium and large follicle counts and the diameter of the dominant follicle were found; however, these results need to be interpreted as they are dependent on the estrus cycle phase of each animal at the scanning day. Otherwise, the number of small follicles is related to the number of follicles present in the ovaries, reflecting the number of remaining primordial follicles and, thus, the ovarian reserve [33]. The lack of differences in numbers of large follicles at 9.5 months of age, before the mean age at puberty (12 months of age), agrees with our finding that CONTROL and SUBNUT heifers reached puberty at a similar age. At 15.5 months of age, the number of small follicles was higher in CONTROL than in SUBNUT heifers. The differentiation of primordial follicles in the ovaries of the female fetus occurs between days 90 and 140 of gestation [9]. A restricted maternal environment in early pregnancy may affect this process, impairing the ovarian reserve [9,34,35]. Mossa et al. [36] described lower antral follicle count in calves born to 60% energy-restricted cows before conception to the end of the first trimester. Maternal nutrient restriction during early gestation reduces the proliferation of ovarian germ-cells and alters the expression of genes that regulate apoptosis in the follicle and granulosa cells, both mechanisms contributing to reduce the number of ovarian primordial follicles [37]. Additionally, we hypothesize that the differences found in IGF-1 concentrations between CONTROL and SUBNUT cows during early pregnancy, which we observed in an earlier phase of this study [21], could have resulted in subsequent changes in fetal hormone levels and in its metabolic pathways, which in turn may have altered gene expression in fetal gonads [38]. Several studies associated a high antral follicle count with improved reproductive efficiency [9,25,35,39], whereas a lower follicle count was related to a reduced lifetime reproductive capacity [40]. In our study, maternal subnutrition could have triggered a disruption in normal gonadal development, reducing the antral follicle count; however, it did not impair the pregnancy success. The ovary diameter was not affected by maternal nutrition in our study. In contrast, Wilkins et al. [41] described lower ovarian weights in heifers from undernourished dams from 80 days of pregnancy to parturition. Regarding the breed, PA heifers had higher numbers of large follicles and bigger ovaries than PI at 9.5 months of age, which agrees with our results that PA heifers reached puberty one month earlier than PI, although this difference was not significant. Parda de Montaña heifers had also larger ovary size than PI at 15.5 months of age, in line with the larger body size measures in PA than in PI heifers.

In the current study, 80% of heifers were pregnant with a single AI, which is a higher pregnancy rate than reported other studies using a similar synchronization protocol [30] or cosynch protocols [42]. This high pregnancy rate could be associated with the optimal body development of heifers at AI (approximately 70% of the mature LW). Gasser [43] recommended breeding for the first time when heifers have reached the 65% of their expected mature LW. Maternal undernutrition did not impair the fertility rate to a single AI. To the best of our knowledge, few studies have reported the effect of the plane of maternal nutrition in early gestation on the fertility rate of the progeny. Martin et al. [23] and Cushman et al. [12] reported that increasing maternal nutrient intake during late gestation improved the pregnancy rate of their daughters. Based on our results, we partially reject our initial hypothesis because no immediate effects were observed on heifer reproductive performance. This could be because the maternal nutrient restriction applied in our study during the first third of pregnancy was not sufficient to induce changes in heifer fertility at first breeding; however, more studies are needed to assess the long-term consequences of a reduced antral follicle count on their lifetime reproductive performance. Regarding the breed, no differences in fertility rate were found between PA and PI heifers in our study, in agreement with previous studies conducted with the same breeds [30].

Surprisingly, in the current study, the ratio of male to female calves was not balanced in SUBNUT and PI heifers, with three times more female than male calves being born, although the differences were not significant. Some authors agree with the Trivers and Willard [44] sex ratio theory, which states that mothers that experienced severe environmental shocks, such us undernutrition, give birth to female calves. This mechanism could protect those fetuses having greater chances of being reproductively successful [45,46,47]. At the beginning of this study, SUBNUT dams had a negative energy balance during the first third of the heifer gestation and PI breed was more sensitive to undernutrition than PA [15]. However, studies involving more animals are needed to demonstrate the sex ratio theory in the progeny.

### 4.3. Heifer Metabolic and Endocrine Profiles

The heifer metabolic and endocrine profiles during rearing exhibited long-term consequences of maternal nutrition, which modified the physiological response of the offspring. Interbreed differences between PA and PI were evidenced.

Glucose profiles are strongly linked with the short-term effect of the current energy and/or protein intake [30]. Furthermore, prenatal nutrient availability may influence the ability of calves to regulate glucose blood concentration during postnatal growing [48]. In our experiment, the lack of differences in glucose concentrations throughout rearing between CONTROL and SUBNUT heifers indicated that glucose metabolism was not affected in their postnatal lives. Similarly, other studies have described no effect of nutritional restriction during early pregnancy on calf basal glucose concentration [11]. Regarding the breed, the higher glucose concentration at AI in PA than in PI could suggest that PA could have consumed more hay and straw (which were offered ad libitum, unlike the concentrate) than PI. This would be consistent with the higher LW and body size measures evidenced at AI.

The NEFA concentrations of SUBNUT heifers were higher than those of the CONTROL group, especially at 13 months of age, around puberty onset. We hypothesize that the higher NEFA concentration in SUBNUT heifers may be due to the extra metabolic effort that allowed SUBNUT heifers to reach a similar LW to that of CONTROL heifers at puberty and breeding. These differences at 13 months of age could also be related to the higher number of large follicles found in CONTROL heifers at this time. According to previous studies [49,50,51], an increase in NEFA concentration may inhibit the granulosa cell survival and proliferation, inducing a reduction in the growth rate of dominant follicles and in the estradiol production. Breed did not affect NEFA metabolism, since concentrations were similar throughout rearing.

Urea concentration tended to be higher in SUBNUT than in CONTROL heifers, especially around puberty onset. Plasma urea concentration is positively correlated with dietary protein intake [52,53], but also with the catabolism of body protein in periods of energy shortfall [30]. The higher requirements in SUBNUT than CONTROL heifers could force heifers to catabolize amino acids from tissue proteins, increasing the urea concentration [54]. In the current study, urea concentration was strongly related to the ADG. Likewise, PA had higher urea concentration than PI which agrees with their higher growth rates.

Cholesterol concentration was higher in SUBNUT than in CONTROL heifers around puberty onset. Cholesterol concentration is positively related with nutrient intake [55]. In our study, cholesterol levels may be associated to the higher liver metabolic rates of SUBNUT heifers, increasing the secretion of very-low-density lipoproteins to blood which increased plasma cholesterol concentrations [56]. Urea and cholesterol concentrations were positively related in the current study. Cholesterol is a precursor for steroid hormone synthesis [57]; therefore, Anderson et al. [58] suggested that an increase in serum cholesterol may influence the reproductive development. However, in our study, no differences in age or LW at puberty onset were found.

Nutritional restriction during the fetal development might affect the IGF axis [48]. In a previous phase of this study, heifers from nutrient-restricted cows had lower IGF-1 concentrations than heifers from control cows at birth [15]. A transgenerational relationship was described between IGF-1 concentrations of cows during early gestation and those from heifers during their suckling period [18]. However, no differences were found between CONTROL and SUBNUT heifers during rearing. The optimal feeding level and growth rate of SUBNUT heifers during rearing may have been reflected in increased IGF-1 concentrations, balancing the IGF-1 values between CONTROL and SUBNUT heifers. Similarly, Maresca et al. [48] described lower IGF-1 concentrations at birth in calves whose mothers had received a low-protein diet from mid-gestation to parturition, with these differences disappearing during calf postnatal growth. Insulin-like growth factor 1 is related to skeletal and muscle development in growing cattle [59] and higher serum IGF-1 concentration is associated with faster growth rates [60]. In our study, IGF-1 concentration was highly correlated with ADG and age at puberty (*r* = −0.7), confirming that IGF-1 is an important mediator involved in the onset of puberty in cattle [61] and could be a good indicator to estimate the age at puberty. The mean IGF-1 concentration of heifers in earlier ages (0–4 months) was moderately correlated with the IGF-1 concentration of heifers during rearing (4–16 months), and to the IGF-1 concentration of their mothers during the first third of gestation (maternal nutritional treatment). No differences were found in IGF-1 concentration between breeds, which agrees with the results reported by Álvarez-Rodríguez et al. [16] and Rodríguez-Sánchez et al. [30].

## 5. Conclusions

Maternal undernutrition in early gestation had some long-term effects on female offspring postnatal performance. Heifers born from underfed cows were lighter at the beginning of the rearing period with this difference disappearing at the end of rearing and during the following gestation and lactation periods. Consequently, heifers from underfed cows increased their nutrient requirements for growth and their metabolic status was impaired during the rearing period. Maternal undernutrition influenced the heifer ovarian development with reduced antral follicle count at breeding but did not affect the age and weight at the onset of puberty nor pregnancy success at single artificial insemination (16 months old) or their calves’ weights during the first lactation. As heifers grow until they are five years old, further research is needed to determine if the growth delay at weaning, the impaired metabolism observed during rearing, and the reduced antral follicle count will impact the attainment of adult weight, mature size, and reproductive lifespan in heifers having suffered maternal undernutrition during their early fetal development.

## Figures and Tables

**Table 1:** Chemical composition of feedstuffs used in the experiment (on an as-fed basis).

Chemical Composition	Total Mixed Ration ^†^	Concentrate ^‡^	Meadow Hay ^§^	Barley Straw ^ф^
DM (g/kg)	908	907	886	902
CP (g/kg DM)	124	152	154	40
NDF (g/kg DM)	466	262	569	796
ADF (g/kg DM)	253	62	320	456
ADL (g/kg DM)	40	8	58	58
Ash (g/kg DM)	113	60	98	65
ME (MJ/kg DM)	11.0	14.4	9.8	7.5

^†^ Diet used during cow gestation (maternal nutrition treatment) and lactation and heifer lactation; ^‡, §, ф^ diet used during heifer rearing; ^§^ diet used during the last month of heifer pregnancy; DM, dry matter; CP, crude protein; NDF, neutral-detergent fiber; ADF, acid-detergent fiber; ADL, acid-detergent lignin; ME, metabolizable energy.

**Table 2:** Heifer productive performance and body size measures during rearing according to maternal nutrition and breed.

Item	Maternal Nutrition		Breed		RSD	*p*-Value	
	CONTROL	SUBNUT	Parda	Pirenaica		Maternal Nutrition	Breed
**Heifer performance**							
LW at weaning (kg)	152 ^a^	133 ^b^	147	138	10.3	0.020	0.270
LW at AI (kg)	415	400	420 ^a^	395 ^b^	20.5	0.199	0.036
ADG during rearing (kg/d)	0.741	0.792	0.823 ^a^	0.710 ^b^	0.0757	0.148	0.002
Age at AI (months)	16.0	15.7	15.6	16.1	0.42	0.248	0.056
**Height at withers**							
At 4 months (weaning, cm)	95	92	95	92	2.9	0.156	0.126
At 12 months (cm)	115	113	117 ^a^	112 ^b^	3.3	0.208	0.010
At 16 months (cm)	121	120	124 ^a^	118 ^b^	2.8	0.435	0.001
**Heart girth**							
At 4 months (weaning, cm)	119	115	118	115	5.3	0.060	0.223
At 12 months (cm)	162	158	163 ^a^	157 ^b^	5.5	0.110	0.027
At 16 months (cm)	175	173	178 ^a^	170 ^b^	5.9	0.416	0.006
**External pelvic area**							
At 4 months (weaning, dm^2^)	9.6	8.7	8.8	9.5	1.07	0.146	0.242
At 12 months (dm^2^)	18.3	17.5	18.7 ^a^	17.1 ^b^	1.42	0.343	0.043
At 16 months (dm^2^)	21.9	21.0	22.6 ^a^	20.3 ^b^	1.31	0.208	0.002

CONTROL, heifers from cows fed 100% of their requirements in early pregnancy; SUBNUT, heifers from cows fed 65% of their requirements in early pregnancy; RSD, residual standard deviation; LW, live weight; AI, artificial insemination; ADG, average daily gain. ^a,b^ Means within a row with different superscripts differ significantly (*p* < 0.05).

**Table 3:** Reproductive performance of heifers during rearing according to maternal nutrition and breed.

Items	Maternal Nutrition		Breed		RSD	*p*-Value	
	CONTROL	SUBNUT	Parda	Pirenaica		Maternal Nutrition	Breed
Age at puberty (months)	12.0	12.1	11.6	12.6	1.58	0.905	0.169
LW at puberty (kg)	341	336	350	327	23.8	0.659	0.076
Mature LW at puberty (%) ^†^	59	58	61	56	4.8	0.723	0.055
Puberty reached by 12 months (%) ^‡^	63	50	63	60	-	0.210	0.272
Puberty reached by 16 months (%)	94	89	95	87	-	0.409	0.333
Fertility to a single AI (%)	78.6	81.3	82.4	76.9	-	0.343	0.328

^†^ 580 kg of expected mature LW for both breeds; ^‡^ % of animals that reached puberty before the mean age at puberty reported in each group; CONTROL, heifers from cows fed 100% of their requirements in early pregnancy; SUBNUT, heifers from cows fed 65% of their requirements in early pregnancy; RSD, residual standard deviation; AI, artificial insemination.

**Table 4:** Follicle population, corpus luteum, and ovary size of heifers during rearing according to maternal nutrition and breed.

Items	Maternal Nutrition		Breed		RSD	*p*-Value	
	CONTROL	SUBNUT	Parda	Pirenaica		Maternal Nutrition	Breed
**Small follicles (<5 mm)**							
At 9.5 months (*n*)	8	9	10	7	4.4	0.365	0.217
At 13 months (*n*)	10	10	9	11	4.1	0.964	0.432
At 15.5 months (*n*)	16 ^a^	11^b^	13	14	4.5	0.011	0.418
**Medium follicles (5 < x < 10 mm)**							
At 9.5 months (*n*)	0.8 ^b^	2.5 ^a^	1.8	1.4	1.45	0.019	0.524
At 13 months (*n*)	0.9	1.9	2.1	0.7	1.79	0.234	0.100
At 15.5 months (*n*)	1.4	0.8	0.9	1.3	1.40	0.364	0.637
**Large follicles (>10 mm)**							
At 9.5 months (*n*)	0.8	0.4	0.8 ^a^	0.4 ^b^	0.49	0.108	0.044
At 13 months (*n*)	0.9 ^a^	0.4 ^b^	0.5	0.8	0.57	0.041	0.367
At 15.5 months (*n*)	0.4 ^b^	0.9 ^a^	0.9	0.4	0.51	0.032	0.056
**Dominant follicle diameter**							
At 9.5 months (mm)	11.2	9.5	10.9	9.8	1.69	0.054	0.227
At 13 months (mm)	11.1	10.2	10.9	10.5	3.19	0.544	0.807
At 15.5 months (mm)	10.5	11.4	12.4 ^a^	9.5 ^b^	2.31	0.451	0.017
**Corpus luteum**							
Heifers with CL at 13 months (%)	88	72	84	73	-	0.191	0.246
CL diameter at 13 months (mm)	19.2	17.9	18.6	18.5	4.25	0.601	0.968
Heifers with CL at 15.5 months (%)	94	83	95	80	-	0.282	0.186
CL diameter at 15.5 months (mm)	13.2	17.2	16.6	13.8	4.13	0.119	0.265
**Ovary diameter**							
At 9.5 months (mm)	14.0	14.4	15.5 ^a^	12.9 ^b^	1.41	0.639	0.009
At 13 months (mm)	18.6	17.5	18.3	17.7	2.02	0.325	0.608
At 15.5 months (mm)	17.8	18.8	19.9 ^a^	16.7 ^b^	1.73	0.292	0.003

CONTROL, heifers from cows fed 100% of their requirements in early pregnancy; SUBNUT, heifers from cows fed 65% of their requirements in early pregnancy; RSD, residual standard deviation; CL, corpus luteum; ^a,b^ means within a row with different superscripts differ significantly (*p* < 0.05).

**Table 5:** Heifer performance during gestation and first lactation according to maternal nutrition and breed.

Items	Maternal Nutrition		Breed		RSD	*p*-Value	
	CONTROL	SUBNUT	Parda	Pirenaica		Maternal Nutrition	Breed
**Heifer performance**							
Gestation ADG (kg/day)	0.334	0.283	0.298	0.319	0.0969	0.275	0.645
LW at calving (kg)	520	491	516	494	33.0	0.103	0.204
BCS at calving	3.0	3.0	2.8 ^b^	3.2 ^a^	0.16	0.425	0.001
Age at calving (months)	26.4	26.3	26.1	26.6	1.52	0.844	0.584
Calving assistance (%)	26.7	16.7	25.0	18.2	-	0.304	0.338
LW at weaning (kg)	469	452	478	443	42.0	0.445	0.124
Lactation ADG (kg/day)	−0.519	–0.349	–0.373	–0.494	0.2318	0.168	0.323
**Calf performance**							
Male/female calf ratio	8/7	3/9	8/8	3/8	-	0.109	0.163
LW at birth (kg)	35	34	36	33	3.7	0.321	0.134
LW at weaning (kg)	111	105	122 ^a^	94 ^b^	19.4	0.505	0.012
Lactation ADG (kg/day)	0.720	0.680	0.814 ^a^	0.587 ^b^	0.1918	0.684	0.031

CONTROL, heifers from cows fed 100% of their requirements in early pregnancy; SUBNUT, heifers from cows fed 65% of their requirements in early pregnancy; RSD, residual standard deviation; ADG, average daily gain; AI, artificial insemination; BCS, body condition score; calving assistance, assisted (from manual pull to caesarean section) or unassisted calving; LW, live weight; ^a,b^ means within a row with different superscripts differ significantly (*p* < 0.05).
